# Effectiveness of interventions delivered within inpatient perioperative care in adults undergoing surgery: scoping review of systematic reviews

**DOI:** 10.1093/bjsopen/zrag013

**Published:** 2026-03-27

**Authors:** Charlotte Davies, Isobel Read, Penny Sucharitkul, Ronelle Mouton, Robert Hinchliffe

**Affiliations:** Bristol Surgical and Perioperative Care Complex Intervention Collaboration, Translational Health Sciences, Bristol Medical School, University of Bristol, Learning and Research Building, Southmead Hospital, Bristol, UK; North Bristol NHS Trust, Southmead Hospital, Bristol, UK; North Bristol NHS Trust, Southmead Hospital, Bristol, UK; North Bristol NHS Trust, Southmead Hospital, Bristol, UK; Bristol Surgical and Perioperative Care Complex Intervention Collaboration, Translational Health Sciences, Bristol Medical School, University of Bristol, Learning and Research Building, Southmead Hospital, Bristol, UK; North Bristol NHS Trust, Southmead Hospital, Bristol, UK

**Keywords:** perioperative pathway, perioperative strategies, primary outcomes, patient outcomes, comprehensive review

## Abstract

**Background:**

Many perioperative interventions have been developed to improve care and health outcomes for patients. Interventions that are effective, reduce adverse events, and improve patient recovery are hugely important to patients and healthcare systems. This study provides a contemporary overview of the effectiveness of interventions delivered within inpatient perioperative care in adults undergoing surgery.

**Methods:**

A scoping review of systematic reviews (SRs) was performed according to Joanna Briggs Institute methodology and PRISMA-ScR guidelines. The following databases were searched: Medline, Embase, Cochrane Library, Cumulative Index to Nursing and Allied Health Literature, and Physiotherapy Evidence Database, last update 2 December 2025.

**Results:**

In all, 190 SRs were included in the review, incorporating 10 themes: enhanced recovery after surgery (ERAS; 77 SRs, 39%); diet/nutritional (31 SRs; 16%); pharmaceutical (20 SRs, 10.8%); respiratory (15 SRs, 8.5%); ‘other’ (e.g. sleep, body warming and personalized nursing interventions, goal directed haemodynamic and acupuncture therapy) (13 SRs, 7.4%); exercise/physical activity (12 SRs, 6.5%); comprehensive geriatric assessment (CGA; 9 SRs, 4.5%); care bundles (5 SRs, 2.8%); multimodal (5 SRs, 2.8%); and physiotherapy (3 SRs, 1.7%). Key intervention themes showed consistent benefit across a range of surgical specialities. These consisted of: respiratory/aerobic strategies on length of hospital stay (LoS), postoperative complications, and the 6-minute walk test, with little evidence for effect on mortality; diet/nutritional strategies, which had significant benefits with regard to LoS, postoperative complications, and surgical site infections, with little or no effect on mortality; CGA, which had a beneficial effect on mortality, LoS, and activities of daily living, with little evidence of effect on readmission; and ERAS, which showed improvements in LoS, postoperative complications, and morbidity, with less evidence of effect on mortality and readmission across specialities.

**Conclusions:**

Key interventions showed consistent patterns of improvement. Before improving or designing new perioperative interventions, it is important to consider and deliver strategies that have already been evaluated and are effective.

## Teaser

This study provides a contemporary overview of the effectiveness of interventions delivered within inpatient perioperative care to adults undergoing surgery. The scoping review provides much needed clarity in a complex and diverse field, and can valuably inform research priorities to allocate often limited resources and help focus on intervention strategies that are likely to provide the most benefit and have the greatest impact on patients after surgery.

## Introduction

Surgical procedures are common, with over 300 million patients globally undergoing surgery each year^1^. Surgery is one of the most important treatments offered in secondary care to improve a patient’s quality of life and survival^2^. However, it carries risks with the potential for poor outcomes, such as postoperative complications, morbidity, and mortality^3^. In the UK, approximately 10% of patients undergoing surgery are at high risk of complications, accounting for around 80% of postoperative deaths^4^. It is hugely important to patients and the National Health Service (NHS), as well as other healthcare systems, that surgical complications can be prevented or treated early, not only to improve what happens during the surgery itself but also to optimize perioperative care around the time of the operation. The aim of this scoping review was to provide a broad overview of the effectiveness of interventions and components of interventions delivered within inpatient perioperative care to adults undergoing surgery reported in systematic reviews (SRs).

There have been many thousands of studies on perioperative care, with many different types of interventions evaluated. This has resulted in a considerable and overabundance of data that is complex, potentially confusing, and often of low quality. It is important to provide a clearer overview for surgeons and patients of the most beneficial interventions that are supported by high-quality evidence. Scoping reviews provide a comprehensive map or broad overview on an extensive body of literature from the existing evidence base^5^ and are more exploratory than SRs^5,6^. This makes the rationale for using scoping review methodology ideal when exploring a diverse range of complex, heterogeneous interventions, outcome measures, and patient surgical populations. Therefore, the aims of the present study lend themselves to a scoping review approach.

Reviews and SRs of prehabilitation interventions have been under considerable focus over recent years within both adult^7–10^ and frail/elderly populations^11–18^, predominantly in elective settings. Review findings have shown benefits from both unimodal^7–9,15^ and multimodal prehabilitation interventions^8,9^ on a range of postoperative outcomes. A recent SR and meta-analysis on prehabilitation interventions and their components suggests that exercise, nutrition, and multicomponent interventions including exercise in adults preparing for major surgery may reduce complication rates and length of hospital stay (LoS) and improve health-related quality of life (QoL) and physical recovery^19^. Interestingly, NHS England has recently requested that trusts integrate prehabilitation as standard practice into their care protocols, highlighting this as an essential part of perioperative care^20^. The perioperative pathway covers the period of care from the patient’s preparation for surgery (prehabilitation/preoperative), through the duration of surgery (intraoperative), and to recovery from surgery (postoperative)^21^.

Patients receiving elective or scheduled operations often have the opportunity and time to prepare for surgery and undertake prehabilitation strategies to try to improve postoperative outcomes. However, patients admitted for emergency surgery are unlikely to have sufficient time or opportunity to implement prehabilitation strategies due to urgent or possible life-threatening conditions requiring surgery. Therefore, understanding which interventions are also effective when delivered within inpatient perioperative care is similarly important and has not been as well reviewed or summarized within the literature.

## Methods

### Study design

A scoping review of previous SRs (with or without meta-analysis) was performed on interventions delivered partly or completely within the inpatient perioperative care setting in adults undergoing surgery to improve health outcomes. A detailed *a priori* protocol for the scoping review was developed before conducting the full review. The scoping review followed Joanna Briggs Institute guidelines^22^ and was conducted using the scoping review methodological framework^5,6^. The review was reported according to the scoping review (PRISMA-ScR) guidelines^5,23^.

This methodology was chosen to provide an overview of the scope of interventions that have been undertaken and analysed in SRs within inpatient perioperative care to improve postoperative outcomes in adults undergoing surgery. For the purposes of this review, inpatient perioperative care was defined as the period from patient admission into hospital for their index surgery to the time of hospital discharge and was divided into three phases: preoperative, intraoperative, and postoperative. Surgery was defined as patients undergoing a surgical procedure in an operating room. All surgical specialities were included in the review except for certain surgical procedures that are outlined in the exclusion criteria provided in the *supplementary methods*.

### Information sources

To identify potentially relevant systematic reviews, the following electronic citation databases were searched from 2000 to 2025: MEDLINE (Ovid), Embase (Ovid), Cumulative Index to Nursing and Allied Health Literature (CINHAL; EBSCO), Physiotherapy Evidence Database (PEDro), and the Cochrane Database of Systematic Reviews.

### Search strategy

A comprehensive multidatabase search strategy was developed by the reviewer (C.D.) with advice from an experienced University of Bristol medical subject librarian. To create the search strategy, the construction of key search terms was based on the three concepts/question framework of Population, Concept, Context, as recommended for scoping reviews^5,6^. Free-text and controlled search terms (Medical Subjects Headings) were used with Boolean operators ‘OR’, to correlate the terms within the same concept, and ‘AND’, which was used to combine searches across the three concepts, with an SR filter. All database searches were run on 28 June 2024 and updated on 2 December 2025. The final search strategy for MEDLINE and the adapted searches for the other databases are shown in the *supplementary results*.

### Eligibility criteria

Eligibility criteria were established using the Population, Concept, Context framework as follows:

Population/participants: adult surgical population (aged ≥ 18 years) undergoing elective or emergency surgery
Concept: interventions delivered in adult surgical patients to improve health outcomes after surgery
Context: interventions or components of interventions delivered partly or exclusively within inpatient perioperative care within the hospital setting (from hospital admission to discharge).

SRs of surgical interventions had to meet the inclusion criteria at each surgical stage of the perioperative pathway; these are outlined in detail in the *supplementary methods*.

### Outcomes

The focus of the review was on primary outcomes reported in SRs, to focus on the most important and strongest evidence. Primary outcomes were recorded if they were related to health outcomes (for example, mortality, postoperative complications), clinical outcomes (for example, LoS, readmission), and patient experience outcomes or patient-reported outcomes related to health (for example, pain, QoL). If an SR reported more than one primary health outcome, all were recorded. Articles were not included if the primary outcomes were not related to a health outcome. If not reported as primary outcomes, the main outcomes were recorded. Data were not extracted for any secondary outcomes measured.

### Screening and study selection process

The final search results were exported into Endnote 21 software^24^ and screened using the predefined eligibility criteria. Any duplicates were removed. Titles and abstracts were independently screened (C.D. and I.R.), and any disagreements were resolved by a third reviewer (P.S.). Only studies with a relevant title and abstract that met the eligibility criteria underwent full paper review. Full texts were reviewed independently by one reviewer (C.D.) for inclusion, with any uncertainties discussed within the reviewing team (P.S., R.H.) to determine final inclusion.

### Data charting process

A data charting form was jointly developed by the study team and was based on Joanna Briggs Institute guidelines for conducting scoping reviews^22^. Data charting was implemented using REDcap software, a customizable electronic information data capture web-based tool^25^. Data charting was undertaken by two reviewers (C.D., I.R.) from eligible studies and initial charting discussed with the study team through pilot testing. Pilot testing involved extracting data from the first five studies using the data charting form and meeting with the study team to determine whether the approach to data charting was consistent with the research question and whether all relevant data were extracted. Further details of information collected on the data charting form are outlined in the *supplementary methods*.

### Dealing with overlapping studies

Included SRs from similar surgical populations were compared to ascertain the degree of overlap between primary studies. If SRs included > 50% of the same studies but reported different primary outcomes, they were still included and underwent data extraction to maximize information gathered on the primary outcomes measured.

## Results

### Study selection

**Fig. 1:**
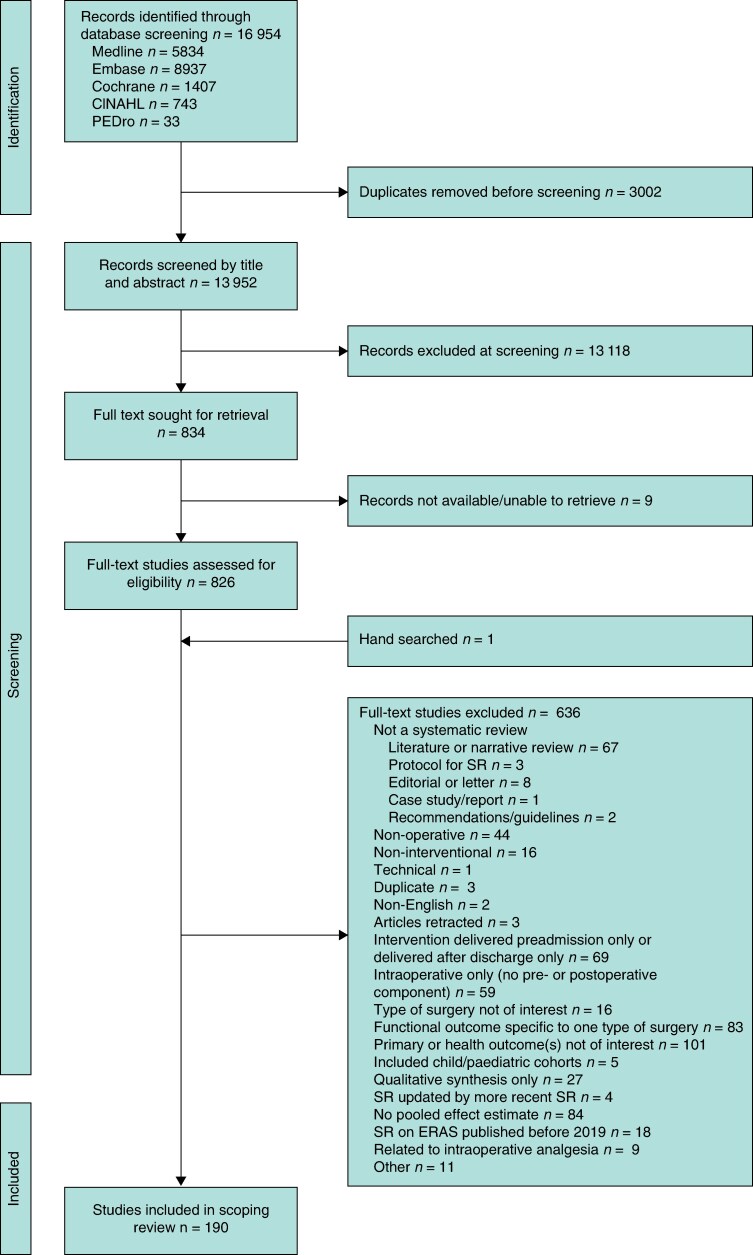
PRISMA-ScR flow diagram of study selection CINHAL, Cumulative Index to Nursing and Allied Health Literature; PEDro, Physiotherapy Evidence Database; SR, systematic review; ERAS, enhanced recovery after surgery.

In all, 16 954 studies were identified from the multidatabase search. Following the removal of duplicated studies, 13 952 were screened to assess eligibility. Based on the title and abstract, 13 118 were excluded, with 834 full-text articles to be retrieved and assessed for eligibility. Of these, 636 articles were excluded. The remaining 190 SRs were considered eligible for inclusion in this review and underwent data charting. The study selection process is shown in the PRISMA-ScR flowchart (*Fig. 1*).

### Characteristics of included SRs

The 190 selected SRs examined the effectiveness of interventions delivered partly or completely within inpatient perioperative care on primary outcomes in adults undergoing surgery. A summary table (*Table 1*) highlights key intervention themes found in the scoping review on the effectiveness of primary outcomes measured across different surgical specialities. *Table 1* shows the primary outcomes with significant beneficial effect and those with no significant difference or not enough evidence for the different intervention themes delivered. The characteristics of the included SRs are provided in *Tables S1–S4* grouped by intervention theme and intervention delivery timepoint, and report the following: first author, publication year, type of surgical speciality and setting, number and type of studies/design, total number of patients/participants, intervention description, and the health-related primary outcome(s) reported in the SR that showed a significant improvement/benefit.

**Table 1:** Summary table highlighting key intervention themes on the effectiveness of primary outcome(s) measured across different surgical disciplines

Intervention theme	Intervention delivery timepoint*	Type of surgical speciality	Effects of intervention(s) on main primary outcome(s) across different surgical specialities from included SRs	
			Significant beneficial effect	No significant difference/not enough evidence
Respiratory/aerobic	Post	Card, Ab, Diff SS		Mortality
	Post	Card, Lung		LoS
	Post	Card, Lung, Ab, Diff SS		Postoperative complications
	Post, Pre and Post	CABG, Pul, Lung, Diff SS	LoS	
	Pre	CABG, Lung, Ab, Diff SS	Postoperative complications	
	Post	CABG, Card	6MWT	
Diet/nutritional	Pre Intra Post, Post	Col, HF, GI, Oncol, Card, Pan, Major surgery		Mortality
	Pre and Post, Pre and/or Post	HF, Card		LoS
	Pre or Post, Post, Pre	HF, GI, Diff SS		Postoperative complications
	Pre, Pre and Post, Post	Col, HF, GI, Card, Oeph, Neuro, Non-card	LoS	
	Pre and Post, Post	Col, HF, GI, Pan, Vis, Oeph	Postoperative complications	
	Pre and Post, Post	Col, Ab, GI, Diff SS	SSIs	
Physiotherapy	Pre, Post or Periop, Post	Diff SS, Card		LoS
	Post	TKA	LoS	
Comprehensive geriatric assessment	Pre Intra Post	HF and FF, HF, Non-card		Readmission
	Pre Intra Post	Orth and Non-orth, Diff SS, HF, HF and FF	Mortality	
	Pre Intra Post	Gen, HF, HF and FF, Non-orth	LoS	
	Pre Intra Post	HF	ADL	
	Pre Intra Post	Non-card, HF	Delirium	
Enhanced recovery after surgery	Pre Intra Post	Col, Liv, GI, Cyto, Oeph, Rad cys, Vas, Pan, HF, Neph, Card, GI		Mortality
	Pre Intra Post	Col, Liv, GI, Bar, Gast, Rad cys, Sp, Pan, HF, THA/TKA, Oeph		Readmission
	Pre Intra Post	Gyn, GI, Cyto, Ab recon, Oeph		Morbidity
	Pre Intra Post	Gyn, Oeph, Bar, Rad cys, Rad pros, Sp, Pan, THA/TKA		Postoperative complications
	Pre Intra Post	Gyn/Onc, GI, Pan, HF		LoS
	Pre Intra Post	All surg, Emer, Gyn/Onc, Col, Liv, Ab, Cyto, Pep, Oeph, Bar, Gast, Rad cys, Thy/Para, Vas, Rad pros, Card, Lung, Sp, Pan, HF, THA/TKA, Neph, Lap, GI	LoS	
	Pre Intra Post	Col, Liv, Digest, Pep, Gast, Rad cys, Vas, Rad pros, Lung, Sp, Pan, HF, THA/TKA, Lap, GI, Pan	Postoperative complications	
	Pre Intra Post	All surg, Col, Ab, Oeph, Vas, Pan, Neph	Morbidity	

^*^Intervention delivery timepoints were classified as before (Pre), during (Intra), or after (Post) surgery. SRs, systematic reviews; Periop, perioperative; Card, cardiac surgery; Ab, abdominal surgery; Diff SS, different surgical specialities; Lung, lung cancer surgery; CABG, coronary artery bypass graft; Pul, pulmonary resection; Col, colorectal surgery; HF, hip fracture surgery; GI, gastrointestinal surgery; Oncol, oncology surgery; Pan, pancreatoduodenectomy; Oeph, oesophageal surgery; Neuro, neurosurgery; Non-card, non-cardiac surgery; Vis, visceral surgery; TKA, total knee arthroplasty; Orth, orthopaedic; Non-orth, Non-orthopaedic; Gen, general surgery; FF, femur fracture surgery; Liv, liver surgery; Cyto, cytoreductive surgery; Rad cys, radical cystectomy; Vas, vascular surgery; Neph, nephrectomy surgery; Bar, bariatric surgery; Gast, gastric surgery; Sp, spine surgery; THA, total hip arthroplasty; Gyn, gynaecological surgery; Ab recon, abdominal wall reconstruction surgery; Rad pros, radical prostatectomy; Gyn/Onc, gynaecological oncology surgery; All surg, all surgery; Emer, emergency surgery; Pep, peptic ulcer surgery; Thy/Para, thyroid/parathyroid surgery; Lap, laparotomy; Digest, digestive surgery; LoS, length of hospital stay; 6MWT, 6-minute walk test; SSIs, surgical site infections; ADL, activities of daily living.

Ten diverse intervention themes were identified. The most studied intervention theme was enhanced recovery after surgery (ERAS; 77 SRs, 41%), followed by diet/nutritional (31, 16%), pharmaceutical/drugs (20, 11%), respiratory/aerobic exercise (15, 8%), ‘other’ interventions (13, 7%), exercise/physical activity (12, 6%), comprehensive geriatric assessment (CGA; 9, 5%), care bundles (5, 3%), multimodal/multicomponent interventions (5, 3%), and physiotherapy (3, 2%).

### Populations

Sample sizes in the 190 SRs ranged from 207 to 297 435 adult patients (age ≥ 18 years); eight SRs did not present sample sizes clearly. Twenty-five reviews (13%) explored population subgroups of older/elderly adults only and 165 reviews (87%) explored adult populations in general. SRs studied surgical populations in elective settings only (81 SRs, 43%), elective and emergency settings (14, 7%), and emergency settings only (7, 4%). Eighty-eight SRs (46%) did not report whether surgery was undertaken in an elective and/or emergency setting. Surgical populations studied in SRs were mostly a mixture of different operations by speciality (36, 19%), followed by abdominal (44, 23%), orthopaedic (33, 17%), and cardiac surgery (20, 11%). The comparator in most of the included interventions was usual or traditional care.

### Primary outcomes

In all, 311 individual primary outcome results were extracted from 190 SRs. LoS was the most studied primary outcome (99 SRs, 32%) followed by postoperative complications (77, 25%), mortality/death (60, 19%), readmission (31, 10%), surgical site infections (SSIs)/wound infections (19, 6%), morbidity (16, 5%), and delirium (16, 5%). The primary outcomes measured for each SR, grouped by intervention theme and surgical speciality, are shown as traffic light plots of effectiveness (*Tables S5–S9*), with red indicating significant harmful/detrimental effects, amber indicating no significant difference or not enough evidence, and green indicating significant beneficial effect (based on a significance level of *P* < 0.05).

### Effectiveness of different intervention themes on primary outcomes

#### Respiratory/aerobic exercise

All included SRs involving respiratory/aerobic exercise interventions were delivered within the elective setting, mainly within cardiac, lung, and abdominal surgeries. None of the included SRs undertook interventions in emergency settings. Approximately half the SRs showing significant improvements on primary outcomes were interventions delivered before and after surgery, with the remaining reviews investigating postoperative interventions only (*Table S1*). SRs mainly investigated either inspiratory muscle training (IMT) or incentive spirometry (IS). Primary outcomes mostly focused on mortality, LoS, the 6-minute walk test (6MWT), and postoperative complications (respiratory and pulmonary complications). Significant positive impacts could be seen, with improvements in LoS, postoperative complications, and the 6MWT across most of the included SRs (*Table S5*). However, none of the included SRs showed any improvements of the intervention on mortality. Pre- and postoperative IS and IMT significantly reduced LoS for patients undergoing lung, cardiac and upper abdominal surgeries. Postoperative complications (for example, respiratory and pulmonary complications) were also significantly reduced in patients undergoing cardiac surgery (preoperative IMT only)^26^, lung cancer surgery (perioperative IS and breathing exercises)^27,28^, and pulmonary, cardiac, and upper abdominal surgery^29^. Postoperative continuous positive airway pressure^30^ and postoperative respiratory or mobilization interventions^31^ also significantly reduced respiratory and pulmonary complications, respectively, within abdominal surgery (*Table S1*).

#### Physiotherapy

SRs on physiotherapy interventions all measured LoS as a primary outcome and all predominantly within the elective setting (*Table S5*). A significant improvement in LoS was only found in one SR^32^, which investigated postoperative delivery of accelerated physiotherapy regimens involving early mobilization in patients undergoing total knee arthroplasty (*Table S1*). SRs of hospital inpatients receiving additional out-of-hours physiotherapy across different types of surgeries^33^ and perioperative experimental physiotherapy interventions initiated in the intensive care unit after cardiac surgery^34^ showed no significant difference in LoS compared with usual care (*Table S5*).

#### Pharmacological/drug interventions

SRs studied pharmaceutical interventions across a wide range of different surgical specialities, with the majority conducted in elective settings. One SR investigated both emergency and elective settings within cardiac surgery^35^. SRs mainly studied preoperative interventions alone or interventions across the preoperative, intra-operative, and postoperative timepoints. The most common primary outcomes measured across studies were mortality, postoperative complications, and delirium, with significant improvements seen across these three primary outcomes in some of the included SRs (*Table S1*).

Preoperative steroids (in hip fracture surgery^36^) and preoperative melatonin or ramelteon (in cardiothoracic, orthopaedic, or hepatic surgeries^37^) had significant effects in reducing the incidence of delirium. Preoperative dexamethasone injection showed significant improvement in LoS for patients undergoing hip fracture surgery^38^.

SRs of interventions delivered at the preoperative, intra-operative, and postoperative timepoints showed that administration of dexmedetomidine (patients aged > 65 years) in non-cardiac surgery^39^ and the administration of dexmedetomidine and melatonin^40,41^in patients undergoing cardiac surgery had significant effects in reducing the incidence of delirium and postoperative delirium. Complications (vasodilatory shock and new-onset atrial fibrillation) were significantly reduced in patients undergoing arginine vasopressin infusion (elective and emergency cardiac surgery)^35^, and reductions in pulmonary complications were seen with prophylactic corticosteroids in cardiac surgery^42^. Significant detrimental and harmful effects were seen with perioperative cerebral oxygen desaturation, with an increase in postoperative delirium (when administered before surgery) in elderly patients undergoing different types of surgery^43^.

#### Exercise/physical activity

Much of the evidence from SRs on exercise/physical activity interventions comes from studies in which the intervention was delivered after surgery, mainly within elective orthopaedic hip fracture and cardiac surgery. None of the included SRs had data from an emergency setting. Significant improvements in mortality rates were seen with postoperative early mobilization in hip fracture surgery^44^. LoS was improved with both early postoperative commencement of physical therapy (lower limb arthroplasty)^45^ and mobilization within 24 hours in patients undergoing lumbar/thoracic surgery^46^ (*Table S1*).

Postoperative complications showed improvements with postoperative early mobilization in hip fracture surgery^44^ and mobilization within 24 hours in lumbar/thoracic surgery^46^. Improvements in mobility were observed with postoperative mobility training^47^ and geriatric team rehabilitation^48^ in patients undergoing hip fracture surgery. For studies measuring 6MWT, interventions consisting of early mobilization and inpatient cardiac rehabilitation in patients undergoing coronary artery bypass grafting^49,50^ showed beneficial effects. Activities of daily living (ADL) were also improved in hip fracture patients receiving postoperative geriatric team rehabilitation^48^. However, postoperative inpatient rehabilitation was associated with a significantly higher risk of complications (periprosthetic) and readmissions compared with home discharge in patients undergoing hip or knee arthroplasty^51^.

#### Diet/nutritional interventions

SRs of diet and nutritional interventions were undertaken across a wide range of elective surgical specialities, predominantly within colorectal, abdominal/gastrointestinal (GI), and orthopaedic surgeries. One SR studied both elective and emergency surgeries^52^. The main impacts on primary outcomes were improvements in LoS, postoperative complications, and SSIs (*Tables S2* and *S7*). Most SRs looked at studies delivered after surgery only or before and after surgery. Four SRs looked at preoperative interventions; these included prebiotics and probiotics (elective liver transplant)^53^ and preoperative carbohydrate treatment/loading (mix of elective surgeries)^54–56^, which showed a positive improvement on infection rate^53^ and LoS^55,56^.

For interventions delivered after surgery, postoperative early oral feeding or early enteral nutrition improved LoS (lower and upper GI surgery)^57,58^, mortality (lower GI surgery)^59^, and pulmonary complications (oesophageal cancer surgery)^60^. The administration of postoperative supplements such as ω-3 fatty acid and selenium in GI cancer surgery and cardiac surgery, respectively, improved LoS^61,62^ and infectious complications^61^. SRs investigating the pre- and postoperative delivery of diet/nutritional interventions mainly consisted of either probiotic/synbiotics, immunonutrition, or nutritional therapies/oral nutritional supplements. Generally, these interventions improved LoS, SSIs, and infectious and postoperative complications. None of the included SRs on diet/nutritional interventions showed any detrimental/harmful effects of the intervention on any of the primary outcomes measured (*Tables S2* and *S7*).

#### ‘Other’ interventions

SRs included within this theme involved interventions that were predominantly delivered within elective settings, with two SRs incorporating both elective and emergency settings^63,64^. The main primary outcomes measured were mortality, LoS, postoperative complications, delirium, and SSIs. The main positive impacts on outcomes were restricted to postoperative complications, the incidence of delirium, and SSIs.

Interventions showing a significant improvement in postoperative complications consisted of goal-directed haemodynamic therapy (any elective or emergency surgeries), personalized nursing (hepatobiliary surgery), rapid rehabilitation nursing (thoracoscopic lung cancer surgery), and protocolized perioperative interventions (non-cardiac surgery). An SR investigating pre- and postoperative sleep interventions (for example, sleep promotion: sensory interventions and dexmedetomidine) and circadian interventions (for example, melatonin and timed bright light) showed that these interventions significantly reduces the incidence of delirium across different surgical procedures^65^. Pre-, intra- and postoperative delivery of acupuncture therapy showed significant improvements in the incidence of postoperative delirium in a mixture of surgical disciplines (orthopaedic, digestive, cardiac and thoracic)^66^ (*Table S2*).

#### Comprehensive geriatric assessment

Evidence from SRs investigating the effectiveness of CGA interventions has predominantly come from studies focusing on elective orthopaedic surgical populations^67–70^ and more broadly within non-cardiac and non-orthopaedic surgeries in both elective and emergency settings^71–73^ (*Tables S3–S5*). Among the primary outcomes investigated, mortality, LoS, readmission, and ADL were most frequently explored across included studies. Noticeable significant improvements in mortality, LoS, and ADL were seen with CGA. Three SRs^69,70,72^ showed that CGA made little difference or no difference to readmission rates when measured as a primary outcome in hip and femur fracture and non-cardiac surgeries. An umbrella review^74^ of SRs that investigated orthopaedic and non-orthopaedic surgeries in both elective and emergency settings (31 SRs, approximately 300 000 elderly patients) showed evidence within the emergency setting that CGA could lower mortality risk at 12 months and could decrease LoS in general surgery by approximately 2 days. The umbrella review^74^ also reported that CGA in hip fracture patients could significantly reduce the risk of delirium and improved mobility and ADL compared with usual/standard care.

#### Multimodal/multicomponent interventions

SRs of multimodal interventions were undertaken across a wide range of surgical specialities, all within elective settings and undertaken across pre-, intra- and postoperative pathways. Most SRs of multimodal interventions measured the incidence of delirium and postoperative complications as primary outcomes, with three of four SRs showing a significant impact of the interventions in reducing postoperative delirium^75–77^. Multicomponent interventions consisting of antipsychotics, bispectral index-guided anaesthesia, and dexmedetomidine treatment successfully reduced the incidence of postoperative delirium in elderly patients undergoing elective non-cardiac surgery^75^. Perioperative geriatric consultations with multicomponent interventions and lighter anaesthesia showed a significant decrease in the incidence of postoperative delirium in non-cardiac surgery patients^76^. Multicomponent interventions (pharmaceutical and non-pharmaceutical) reduced the incidence of delirium compared with usual care in patients undergoing surgery (majority orthopaedic surgery)^77^. Clinical care pathways^78^ and patient blood management programmes^79^ improved in-hospital complications and total complications, respectively in different types of surgery (*Table S3*).

#### Care bundle interventions

SRs of care bundle interventions were undertaken across a range of surgical specialities; two reviews within elective settings^80,81^, one in elective and emergency settings^82^, and two in emergency settings only^83,84^. Most care bundle interventions were delivered across pre-, intra- and postoperative pathways. Interventions undertaken within the emergency setting showed no significant effect on mortality, readmissions (emergency abdominal and general surgery), or reoperations (emergency abdominal surgery) compared with usual care. However, in emergency abdominal surgery, care bundle interventions did show significant improvements in LoS, postoperative complications, and SSIs^83^. These interventions consisted of multimodal perioperative care bundles, perioperative protocols, and dedicated clinical pathways delivered to all patients in a standardized manner. Within elective settings, the interventions consisted mainly of those designed specifically to prevent SSIs, and were shown to result in significant reductions in SSIs at 30 days after colorectal surgery^80^ and in the incidence of SSIs in different types of surgery (time series studies only)^81^ (*Table S3*).

#### ERAS protocols

Most evidence for the use of ERAS protocols comes from studies undertaken in elective settings, with only five SRs conducted within emergency settings^85–89^. Positive impacts on outcomes were mainly seen with improvements in LoS, morbidity, postoperative complications, SSIs/wound infection, and pain across elective surgical specialties. Generally, ERAS protocols made no significant difference to mortality and readmission rates when measured as primary outcomes across surgical specialities. However, the ERAS protocol was shown to significantly reduce mortality in orthopaedic surgery^90^ and gastrectomy^91^. SSIs/wound infections were significantly reduced using ERAS protocols within colorectal^92^, peptic ulcer^93^, gastric cancer^91^, lung^94^, laparotomy^89^, and hip fracture surgery^95^ (*Table S4*). ERAS protocols undertaken within emergency settings showed significant improvements in LoS in four of five SRs. However, ERAS protocols may not be beneficial in all surgical specialities, with significant harmful/detrimental effects observed in terms of morbidity and readmission rates in patients undergoing gastric cancer surgery undertaken in an umbrella review of SRs^90^ and increased major complications observed in patients undergoing bariatric surgery^96^ (*Table S9*).

## Discussion

This scoping review aimed to identify and provide a comprehensive overview from available research evidence to date from SRs of interventions delivered within inpatient perioperative care in adults undergoing surgery and to provide a clear pattern of the most effective interventions. To the best of the authors’ knowledge, this is the first scoping review to summarize this extensive body of literature. Much focus has been on prehabilitation in the phase before admission to hospital. However, the present study has looked at interventions delivered fully or partially within hospital where timing and delivery are important for many procedures and specialities that demand an urgent timeline and where there is little opportunity to deliver interventions before patients are admitted to hospital. The review covered both elective and emergency surgeries. However, almost half the included SRs did not report whether studies were undertaken in emergency or elective settings. This lack of information about the time-based categories of the surgical procedures is a limitation that impacts on a more in-depth interpretation of the data.

The review identified some key interventions that showed consistent patterns of improvement on primary outcomes. Significant evidence of effectiveness was evident from both simple strategies such as diet/nutritional, drugs, respiratory/aerobic, and exercise interventions to more complex multicomponent interventions delivered across the patient perioperative pathway such as CGA and ERAS protocols.

Interventions within the theme of diet/nutrition showed encouraging evidence predominantly within elective surgical specialities. These included pre- and postoperative delivery of nutritional supplements (for example, prebiotics, probiotics, synbiotics, and immunonutrition/nutritional therapies), with consistent patterns of improvement in LoS, postoperative complications, and SSIs. After surgery, early oral feeding and early enteral nutrition generally improved LoS, mortality, and pulmonary complications. There was little or no difference observed in mortality. Effective respiratory/aerobic interventions were observed mainly with IMT and/or IS predominantly delivered either after surgery or before and after surgery across different elective surgical specialities (mainly cardiac, abdominal, and lung surgeries). Findings showed consistent patterns of improvements in LoS and postoperative complications. Results of the 6MWT improved significantly with postoperative IMT and postoperative immediate aerobic exercise in cardiac patients. As for the diet/nutrition theme, respiratory/aerobic interventions showed little evidence for any change or effect on mortality across different surgical populations.

For exercise/physical activity interventions, effective strategies were mainly delivered after surgery within inpatient care and focused on early mobilization, early physical therapy, and inpatient rehabilitation within cardiac and orthopaedic surgeries. Consistent improvements were seen in LoS, postoperative complications, 6MWT, and mobility. Current recommendations advocate early mobilization or early ambulation over prolonged bed rest after orthopaedic surgery^97^, and early mobilization is a core component of ERAS programmes that require patients to become as active as early as possible on the day of or day after surgery^98^. Review findings add to the evidence base that early mobilization after surgery is an important postoperative intervention that can improve patient outcomes.

More complex multicomponent interventions, such as CGA, were also shown to be beneficial with regard to patient primary outcomes. CGA is applied to older geriatric surgical populations and involves diagnostic and treatment methods that identify medical, psychosocial, and functional weaknesses of an elderly person to develop a coordinated multidisciplinary plan to help achieve better health outcomes^99^. Evidence from SRs of CGAs showed a predominant effect on mortality in both elective and emergency settings and CGA was a noticeable benefit to this important outcome compared with all other types of interventions included in the review. There were little or no significant effects observed on readmission rates across CGA reviews. Multicomponent ERAS programmes also showed consistent advantages for patients across a range of surgical disciplines, with positive impacts seen mainly on LoS and postoperative complications. Generally, no significant effects were seen on mortality and readmission. ERAS protocols use multidisciplinary and patient-centred approaches to optimize patient care through the perioperative pathway to aid postoperative recovery^100^. These programmes have been widely studied across surgical specialities over the past 10 years due to evidence of benefit since ERAS protocols were first applied in colorectal surgery^101^. Due to a recent umbrella review undertaken in 2020 on ERAS protocols in different surgical specialities^90^, the review only included ERAS studies from 2019 onwards. Zhang *et al*.^90^ found ERAS to be safe, feasible, and efficient across most surgical specialities, especially within orthopaedic and spinal surgery. The additional data from 2019 onwards of a further 77 SRs on the effectiveness of ERAS protocols across different surgeries adds to this positive evidence base. These recent SRs show a small number of ERAS programmes or modified versions of ERAS having been undertaken in emergency settings; these included laparotomy^89^ and colorectal cancer surgery^86^ and intra-abdominal surgery^87^. In laparotomy, ERAS improved LoS, complications and SSIs^89^, in colorectal surgery ERAS improved LoS and overall complications^86^ and for intra-abdominal surgery LoS and morbidity^87^. In a mixture of different emergency surgeries ERAS protocols showed significant improvements in LoS^85^.

The findings of this review reveal that the main primary outcomes reported for measuring the effectiveness of interventions delivered partly or completely within inpatient perioperative care were LoS, postoperative complications, mortality, and readmission. These four outcomes covered approximately 90% of all the primary outcomes reported within the review and appear to be the main indicators currently used by clinicians to evaluate the effectiveness of interventions. However, it is important to note that other different metrics may have been used or reported as secondary outcomes in the SRs (for example, functional status or other patient symptoms) but were beyond the scope of this review for inclusion. Other outcomes that could be used to measure intervention effectiveness, such as 90-day mortality, days alive out of hospital and failure to discharge home, could also serve as important indicators to evaluate intervention effectiveness.

It is clear from these findings that a number of different intervention strategies have positive impacts on different primary outcomes. This information is key for surgeons and healthcare professionals trying to optimize perioperative care for patients within their specific surgical speciality. Knowledge of which interventions are most likely to improve specific patient outcomes is important for ensuring patients receive the highest quality care before, during, or after surgery. Strategies based on high-quality evidence that are associated with a reduced risk of complications and improved patient safety, and that lead to faster recovery, will help guide surgeons to make more informed decisions regarding best treatment choices and more focused delivery of often limited resources so they can best improve patient outcomes.

This review has identified some key gaps. Much of the evidence was generated from elective surgical populations with very small numbers of SRs conducted within emergency settings and within older/elderly cohorts. This finding is not surprising due to the more challenging and unpredictable nature of emergency surgery in terms of generating robust evidence and being able to deliver effective interventions in a busy clinical environment, which is a key barrier to effective implementation. Interestingly, SRs incorporating primary studies within emergency settings predominantly focused on the administration of different pharmaceutical drugs or involved multicomponent interventions such as care bundles, CGA, and ERAS protocols. The nature of the delivery of these types of strategies helps address some of the challenges in these surgical populations. The findings highlight the need and opportunity for future research to focus on intervention development within emergency surgical disciplines where timely interventions are even more crucial.

The review has generated an informative summary on intervention effectiveness but there are some limitations. Only primary outcome data were reported from SRs to concentrate on the most important and strongest evidence, and to make the review as feasible and manageable as possible. The review only included SRs; therefore, the results are based on evidence that may not include newly published primary studies that may have been relevant to the topic. The date and English-language-only restrictions may also have excluded some SRs, leading to potential selection bias. The scoping review included some SRs with overlapping studies, potentially leading to double counting of evidence. Some of the primary studies included in the SRs involved intervention delivery within inpatient perioperative care that also overlapped into preadmission and post-discharge delivery, highlighting the heterogeneity of primary studies, which was particularly evident in respiratory/aerobic themed interventions. It should also be noted that the scoping review provides an overview of the existing evidence on intervention effectiveness regardless of the methodological quality or risk of bias reported in the SRs.

The review has many strengths: it was a considerably large undertaking, and a thorough search was conducted on 28 June 2024 and updated on 2 December 2025. The search strategy ensured the SRs included in the scoping review were based on the most relevant and up-to-date information from the outset. A comprehensive and broad systematic search strategy was used that was applied to five major citation databases. The search generated inclusion of > 100 evidence sources, defining it as a large scoping review^102^. The inclusion of 190 SRs with data synthesized from hundreds of primary studies incorporating > 1600 randomized clinical trials enabled a considerable amount of robust and reliable evidence to be summarized. Using scoping review methodology allowed a comprehensive overview to be undertaken on an extensive body of literature.

This review has mapped key findings and revealed patterns and trends in the data that provide important insights into the current evidence landscape. Before designing new or making improvements to existing surgical interventions, it is important to first consider and deliver those interventions that have already been evaluated and are known to be effective; this will help better inform future surgical intervention development. Understanding which strategies are most successful across different surgical specialities and settings will help surgeons and healthcare professionals optimize and develop their treatments and approaches. This will ultimately improve patient recovery and lead to better surgical outcomes for patients.

## Supplementary Material

zrag013_Supplementary_Data

## Data Availability

The data used in the scoping review has not been archived in a public repository, but the authors will be able to consider specific requests for data sharing on an individual basis.
