# Supplementary material for: Effectiveness of interventions delivered within inpatient perioperative care in adults undergoing surgery: scoping review of systematic reviews
Source: BJS Open. 2026 Mar 27;10(2):zrag013. doi: 10.1093/bjsopen/zrag013 (PMC13023745; doi:10.1093/bjsopen/zrag013)
Supplement: zrag013_Supplementary_Data [file zrag013_supplementary_data.docx]

**An overview on effectiveness of interventions delivered within inpatient perioperative care in adults undergoing surgery: A scoping review of systematic reviews**

Charlotte Davies^1^, Isobel Read^2^, Penny Sucharitkul^2^, Ronelle Mouton^2^ Robert Hinchliffe^1,2^

^1^Bristol Surgical and Perioperative Care Complex Intervention Collaboration, Translational Health Sciences, Bristol Medical School, University of Bristol, Learning and Research Building, Southmead Hospital, Southmead Road, Bristol, BS10 5NB, UK. ^2^North Bristol NHS Trust, Southmead Hospital, Southmead Road, Bristol, BS10 5NB, UK

Corresponding author: Dr Charlotte Davies, Bristol Surgical and Perioperative Care Complex Intervention Collaboration, Translational Health Sciences, Bristol Medical School, University of Bristol, Learning and Research Building, Southmead Hospital, Southmead Road, Bristol, BS10 5NB, UK, ORCID ID: 0000-0002-3468-4258

**Supplementary Materials**

**Supplementary Methods**

**Eligibility criteria for preoperative, intraoperative and postoperative interventions**

**Preoperative interventions**: SRs were included of interventions delivered within the inpatient /hospital admission before surgery in elective and emergency patients. Excluded were any SRs of prehabilitation (preadmission) interventions delivered solely to patients before admission to hospital for surgery. SRs were included if the SR included a mix of pre and postoperatively delivered inpatient perioperative interventions studies.

**Intraoperative interventions**: SRs of intraoperative interventions delivered during surgery were included only if there was a preoperative and/or a postoperative component (e,g ERAS protocols or a bundle of care to reduce surgical site infection) in both elective and emergency settings. Excluded were intraoperative interventions delivered purely in isolation during surgery or directly related to a surgical technique (e.g surgical incision methods, reducing blood loss during surgery, skin closure techniques, wound healing/irrigation). SRs of intraoperative interventions related to surgical analgesia/pain relief or comparison of anesthetic techniques were also excluded.

**Postoperative interventions**: SRs of postoperative interventions were included if delivered after surgery within the inpatient/hospital admission for both elective and non-elective settings. Excluded were any SRs of postoperative interventions initiated and delivered after the patient had been discharged from hospital. SRs were also included if a mix of pre- and post-operative inpatient perioperative intervention studies were included in the SR.

If a SR included a mixture of preadmission, inpatient interventions and post discharge intervention studies, the SR was included if >50% of studies delivered inpatient perioperative interventions. Interventions involving multi component interventions such as enhanced recovery after surgery (ERAS) and comprehensive geriatric assessment (CGA) were also included in the review. Therefore, SRs were included even if some of the components/elements of a multicomponent intervention were delivered before patient admission to hospital or after hospital discharge if some intervention components were delivered during the inpatient hospital admission. This approach was deemed most pragmatic as multicomponent interventions are delivered as a package of care across the perioperative pathway before, during and after hospital admission and SRs are unlikely to report effects on postoperative outcomes based on separate individual components. SRs on ERAS intervention protocols were only included from 2019 onwards due to a recently published umbrella review of SRs in 2020 conducted on ERAS intervention effectiveness across surgical specialties (90)

Additional inclusion criteria were i) Systematic reviews of randomised and non-randomised studies (with or without meta-analysis), ii) Adults only (≥18 yrs) undergoing elective or emergency surgery, iii) In English language only, iv) limited to the date of publication between 2000 and July 2024 v) SRs had to report pooled effect estimates/sizes (e.g odds ratio (OR), relative risk (RR), hazard ratio (HR), mean difference (MD) or standard mean difference (SMD)). vi) reviews of interventions delivered within inpatient perioperative care (preoperatively, intraoperatively or postoperatively or delivered across all 3 stages).

Additional exclusion criteria were i) child/adolescent population (<18yrs) undergoing surgery ii) non-English language reviews, iii) primary research studies, non-systematic searches, conceptual reviews, historical reviews, scoping reviews, narrative reviews, reviews of qualitative studies, clinical summaries theses, dissertations, protocols for systematic reviews, systematic reviews only available as an abstract (e.g conference abstracts). iv) studies conducted in developing countries where context and setting were different to UK/ NHS setting v) specific types of surgery (e.g ophthalmic, ear, dental/oral, hand, facial surgery, obstetric surgery, surgery for infertility, plastic surgery, surgery for sports related injuries, or minor surgery involving local anaesthetic e.g for skin cancer removal) vi) Intervention(s) designed to improve a functional outcome specific to one type of surgery.

If a SR had been updated and newer evidence superseded earlier findings, we used the results from the most recent SR. We limited our eligibility criteria to SR published from 2000 to present to ensure relevance to current/best practice as reporting standards for meta-analysis and SRs were first introduced in 1999.

**Data Charting/Extraction Form**

The data charting form captured information in three main areas; i) Study characteristics (e.g Author, year, title, country of origin, number and types of study design, participant size, study population, age range and/or mean age) ii) Intervention characteristics (e.g intervention description, intervention timepoint (pre, intra, postoperative) intervention details (e.g components, duration and delivery format), intervention theme, surgical specialty, intervention setting, and aim of SR iii) Evidence of intervention(s) effectiveness; primary outcome(s) measured and pooled effect sizes /estimates were recorded (e.g HR, MD, SMD, OR, RR, p values and CIs) and key findings/conclusions extracted. Each primary outcome(s) pooled effect estimate was allocated a Red/Amber/Green (RAG) traffic light colour rating based on its reported level of intervention effectiveness. The final version of the data charting form is shown in the supplementary material.

**Supplementary Results**

**Database search strategies for scoping review**

**Searches run on the 28/6/24 and updated on 2/12/25 on Medline, Embase, Cochrane library, CINAHL and PEDro databases**

**Medline search strategy**

1. exp Specialties, Surgical/

2. exp Surgical Procedures, Operative/

3. surg*.tw.

4. operat*.tw.

5. exp Perioperative Care/

6. exp Perioperative Period/

7. Perioperative.tw.

8. Peri-operative.tw.

9. Preoperative.tw.

10. Pre-operative.tw.

11. (Presurg* or Pre-surg*).tw.

12. Intraoperative.tw.

13. Intra-operative.tw.

14. Postoperative.tw.

15. Post-operative.tw.

16. (Postsurg* or post-surg*).tw.

17. Intervention*.tw.

18. exp Enhanced Recovery After Surgery/

19. ERAS.tw.

20. (enhanc* adj3 recover*).tw.

21. (acceler* adj3 recover*).tw.

22. ("fast track*" or "fast-track*").tw.

23. exp geriatric assessment/

24. "comprehensive geriatric* assessment*".tw.

25. exp Critical Pathways/

26. "care pathway*".tw.

27. exp Patient Care Bundles/

28. "care bundle*".tw.

29. "care package*".tw.

30. exp Early Ambulation/

31. (early adj3 ambulat*).tw.

32. exp Rehabilitation/

33. (prehab* or pre-hab*).tw.

34. (rehab* or re-hab*).tw.

35. (multimodal* or multi-modal*).tw.

36. (multi-component* or multicomponent*).tw.

37. (interdisciplin* or inter-disciplin* or multidisciplin* or multi-disciplin*).tw.

38. 1 or 2 or 3 or 4

39. 5 or 6 or 7 or 8 or 9 or 10 or 11 or 12 or 13 or 14 or 15 or 16

40. 17 or 18 or 19 or 20 or 21 or 22 or 23 or 24 or 25 or 26 or 27 or 28 or 29 or 30 or 31 or 32 or 33 or 34 or 35 or 36 or 37

41. 38 and 39 and 40

42. limit 41 to yr="2000 -Current"

43. limit 42 to (english language and humans)

44. limit 43 to "systematic review"

**Embase search strategy**

1 exp surgery/

2 surg*.tw.

3 operat*.tw.

4 exp perioperative period/ or exp perioperative care/

5 Perioperative.tw.

6 Peri-operative.tw.

7 pre-operative.tw.

8 preoperative.tw.

9 (Presurg* or Pre-surg*).tw.

10 Intraoperative.tw.

11 Intra-operative.tw.

12 Postoperative.tw.

13 Post-operative.tw.

14 (Postsurg* or post-surg*).tw.

15 Intervention.tw.

16 exp enhanced recovery after surgery/

17 "enhanced recovery after surgery".tw.

18 (enhanc* adj3 recover*).tw.

19 (acceler* adj3 recover*).tw.

20 ("fast track*" or "fast-track*").tw.

21 exp geriatric assessment/

22 "comprehensive geriatric* assessment*".tw.

23 exp clinical pathway/

24 "care pathway*".tw.

25 exp care bundle/

26 "care bundle*".tw.

27 "care package*".tw.

28 exp mobilization/

29 (early adj3 ambulat*).tw.

30 exp rehabilitation/

31 (prehab* or pre-hab*).tw.

32 (rehab* or re-hab*).tw. 312137

33 (multimodal* or multi-modal*).tw.

34 (multi-component* or multicomponent*).tw.

35 (multidisciplin* or multi-disciplin*).tw.

36 (interdisciplin* or inter-disciplin*).tw.

37 1 or 2 or 3

38 4 or 5 or 6 or 7 or 8 or 9 or 10 or 11 or 12 or 13 or 14

39 15 or 16 or 17 or 18 or 19 or 20 or 21 or 22 or 23 or 24 or 25 or 26 or 27 or 28 or 29 or 30 or 31 or 32 or 33 or 34 or 35 or 36

40 37 and 38 and 39

41 limit 40 to yr="2000 -Current"

42 limit 41 to (human and english language)

43 limit 42 to "systematic review"

44 limit 43 to conference abstracts

45 43 not 44

46 limit 45 to (embryo <first trimester> or infant <to one year> or child <unspecified age> or preschool child <1 to 6 years> or school child <7 to 12 years>)

47 45 not 46

**Cochrane Library search strategy**

#1 MeSH descriptor: [General Surgery] explode all trees

#2 MeSH descriptor: [Surgical Procedures, Operative] explode all trees

#3 MeSH descriptor: [Specialties, Surgical] explode all trees

#4 surg* or operat*:ab,ti,kw

#5 #1 or #2 or #3 or #4

#6 MeSH descriptor: [Perioperative Care] explode all trees

#7 MeSH descriptor: [Perioperative Period] explode all trees

#8 perioperative or peri-operative or preoperative or pre-operative or presurg* or pre-surg* or intraoperative or intra-operative or postoperative or post-operative or postsurg* or post-surg*:ab,ti,kw

#9 #6 or #7 or #8

#10 Intervention*:ab,ti,kw

#11 MeSH descriptor: [Enhanced Recovery After Surgery] explode all trees

#12 ERAS:ab,ti,kw

#13 enhanc* NEAR/3 recover*:ab,ti,kw

#14 acceler* NEAR/3 recover*:ab,ti,kw

#15 fast track* or fast-track*:ab,ti,kw

#16 MeSH descriptor: [Geriatric Assessment] 5 tree(s) exploded

#17 comprehensive geriatric* assessment*:ab,ti,kw

#18 MeSH descriptor: [Critical Pathways] explode all trees

#19 care NEXT pathway*:ab,ti,kw

#20 MeSH descriptor: [Patient Care Bundles] explode all trees

#21 care bundle* or care package*:ab,ti,kw

#22 MeSH descriptor: [Early Ambulation] explode all trees

#23 early NEAR/3 ambulat*:ab,ti,kw

#24 MeSH descriptor: [Rehabilitation] explode all trees

#25 prehab* or pre-hab* or rehab* or re-hab*:ab,ti,kw

#26 multimodal* or multi-modal* or multi-component* or multicomponent* or interdisciplin* or inter-disciplin* or multidisciplin* or multi-disciplin*:ab,ti,kw

#27 #10 or #11 or #12 or #13 or #14 or #15 or #16 or #17 or #18 or #19 or #20 or #21 or #22 or #23 or #24 or #25 or #26

#28 #5 and #9 and #27

with Cochrane Library publication date from Jan 2000 to Jul 2024, in Cochrane Reviews

**CINAHL search strategy**

S35 S32 AND S33 AND S34

S34 S11 OR S12 OR S13 OR S14 OR S15 OR S16 OR S17 OR S18 OR S19 OR S20 OR S21 OR S22 OR S23 OR S24 OR S25 OR S26 OR S27 OR S28 OR S29 OR S30 OR S31

S33 S4 OR S5 OR S6 OR S7 OR S8 OR S9 OR S10

S32 S1 OR S2 OR S3

S31 TX (multidisciplin* OR multi-disciplin* OR interdisciplin* OR inter-disciplin*)

S30 TX (multicomponent* OR multi-component*)

S29 TX (multimodal* OR multi-modal*)

S28 TX (rehab* OR re-hab*)

S27 (MH "Rehabilitation+")

S26 TX (prehab* or pre-hab*)

S25 (MH "Prehabilitation")

S24 TX early N3 ambulat*

S23 (MH "Early Ambulation")

S22 TX (care bundle* OR care package*)

S21 (MH "Patient Care Plans+")

S20 TX care pathway*

S19 (MH "Critical Path")

S18 TX geriatric* assessment*

S17 (MH "Geriatric Assessment+")

S16 TX (fast track* OR fast-track*)

S15 TX acceler* N3 recover*

S14 TX enhanc* N3 recover*

S13 TX Intervention*

S12 TX Enhanced Recovery After Surgery

S11 (MH "Enhanced Recovery After Surgery")

S10 TX (Postsurg* OR post-surg*)

S9 TX (Postoperative OR post-operative)

S8 TX (Intraoperative OR intra-operative)

S7 TX (Presurg* OR pre-surg*)

S6 TX (Preoperative OR pre-operative)

S5 TX (Perioperative OR peri-operative)

S4 (MH "Perioperative Care+")

S3 TX (Surg* OR operat*)

S2 (MH "Specialties, Surgical+")

S1 (MH "Surgery, Operative+")

Limiters - Publication Date: 20000101-20240731; English Language; Research Article; Human; Publication Type: Systematic Review; Age Groups: All Adult; Language: English

Expanders - Apply related words; Also search within the full text of the articles; Apply equivalent subjects

Search modes - Proximity

**PEDro search**

Abstract & Title: surg* intervention* perioperative

Method: Systematic review

Published Since: 2000

When searching: match all search terms (AND)

**Supplementary Tables**: **Characteristics of included SRs** **by intervention theme and intervention delivery timepoint (Tables S1 to S4)**

**Table S1:** Systematic Reviews of Respiratory/Aerobic, Physiotherapy, Pharmaceutical/Drugs and Exercise/Physical Activity Interventions showing significant benefit on primary outcomes

**Table S1:** LoS; Length of stay, QoL: Quality of Life, PPCs: Post operative pulmonary complications, VT: Venous thromboembolism, Pul comp; Pulmonary complications, Resp comp; Respiratory complications, RCT: Randomised controlled trial, NR: Non randomised trial, CABG: Coronary artery bypass graft, THA: Total hip arthroplasty,

Admin: Administration, LoS: Length of stay, POD: Postoperative delirium, Del: Delerium, Mort: Mortality, MI: Myocardial Ischemia, Retro: Retrospective study, Prospec: Prospective, 6MWT: 6 min walk test, w/e PT: Weekend Physical therapy, del: delayed, ADL: Activities of daily living, LoS: Length of stay, Ad to n/home: admission to nursing home, Postop comp: Postoperative complications, CABG: Coronary artery bypass grafting. * SR did not report if elective &/or emergency surgery

**Table S2:** Systematic Reviews of Diet/Nutritional and 'Other' Interventions showing significant benefit on primary outcomes

**Table S2**: RCTs: randomised controlled trials, C/C: case contol studies, NK: Not known, Supp: Supplements, Infect Comp: Infectious complications, Total comp: Total complications, ICU: Intensive care unit, Blood trans: Blood transfusion, ONS: Oral nutritional supplements, Admin: administration, AAs: Amino acids, GI: Gastrointestinal, LoS: Length of stay, SSI: Surgical site infection, +/-: with or without, PUFAs: polyunsaturated fatty acids, POSH: Perioperative optimisation of senior health, CGA: Comprehensive geriatric assessment, LIFE: Liaison Intervention in frail elderly, LoS; length of stay, SSI: Surgical site infection, RBC: Red blood cell,Phys Funct: Physical Function, Coh St: Cohort studies, Pul comp; Pulmonary complications, PPCs: Postoperative pulmonary complications, NSAIDs: Non-steroidal anti-inflammatory drugs, TENS: Transcutaneous electrical nerve stimulation, p/way: pathway, POD: Postoperative delirium, Incid del: Incidence of delerium, sens anal:sensitivity analysis, S & L/term: Short and long term, ITS: Interrupted time series studies, S/ficial: Superficial, PTCA Percutaneous Transluminal Coronary Angioplasty.* SR did not report if elective &/or emergency surgery

**Table S3:** Systematic Reviews of CGA, Multicomponent and Care bundle Interventions showing significant benefit on primary outcomes

**Table S3** : LoS; Length of stay, QoL: Quality of Life, MI: Myocardial Ischaemia, PPCs: Post operative pulmonary complications, VT: Venous thromboembolism, POD: Postoperative delirium, ICU: Intensive care unit, ED : Emergency Dept, Ad Ev: Adverse event , L/term: Long term, Total Comp: Total complications, Pul comp ; Pulmonary complications, Resp comp; Respiratory complications, Pul & Clin comp: Pulmonary and clinical complications, Periop comp; Perioperative complications, VAS: Visual analogue scale, Mob: mobility, VTE: Venous thromboembolism, CGA: Comprehensive geriatric assessment, CABG: Coronary artery bypass grafting, DEX: Dexamethasone, RCTs: randomised controlled trials, NK: Not known , B & A : Before and after studies, POSH: Perioperative optimisation of senior health, CGA: Comprehensive geriatric assessment, LIFE: Liaison Intervention in frail elderly, LoS; length of stay, SSI: Surgical site infection, RBC: Red blood cell, Phys Funct: Physical Function, Coh St: Cohort studies, PPCs: Post operative pulmonary complications, NSAIDs: Non-steroidal anti-inflammatory drugs, TENS: Transcutaneous electrical nerve stimulation, p/way: pathway, Incid del: Incidence of delerium, sens anal: sensitivity analysis, S & L/term: Short and long term, ITS: Interrupted time series studies, S/ficial: Superficial, PTCA Percutaneous Transluminal Coronary Angioplasty. * SR did not report if elective &/or emergency surgery

**Table S4:** Systematic Reviews of Enhanced Recovery after Surgery (ERAS) / Fast Track Surgery Interventions showing significant benefit on primary outcomes

**Table S4** : RCTs: randomised controlled trials, CC: case/control studies, NR: Non randomised studies, CS: Cohort studies, Obs: observational studies, ERAS: Enhanced recovery after surgery, FTS: Fast track surgery, AA: aortic aneurysm, THA : Total hip arthroplasty, TKA: Total knee arthroplasty, O/paed: Orthopaedic surgery, Gastric C surg: Gastric cancer surgery, Overall comp: overall complications, GI: Gastrointestinal, VAS: Visual analogue scale, OFB amb: Time to out of bed ambulation , POI: postoperative paralytic ileus, EN: Enhancement of nutrition , PPOI: prolonged postoperative ileus. Min Invas: Minimally invasive, LoS: Length of Stay * SR did not report if elective &/or emergency surgery

**Supplementary Tables: Traffic Light Plots of Effectiveness of Interventions on Primary Outcomes (Tables S5 to S9)**

**Table S5: Traffic-light plots to summarise the overall effectiveness of CGA, Respiratory/Aerobic, Physiotherapy and Pharmaceutical/Drug interventions in any type of surgery.**

**Table S5**: Traffic light plots of effectiveness of CGA, respiratory, physio and pharmaceutical interventions. LoS; Length of stay, QoL: Quality of Life, MI: Myocardial Ischaemia, PPCs: Post operative pulmonary complications, VT: Venous thromboembolism, POD: Postoperative delirium, ICU: Intensive care unit, ED : Emergency Dept, Ad Ev: Adverse event , L/term: Long term, Total Comp: Total complications, Pul comp ; Pulmonary complications, Resp comp; Respiratory complications, Pul & Clin comp: Pulmonary and clinical complications, Periop comp; Perioperative complications, VAS: Visual analogue scale, Mob: mobility, VTE: Venous thromboembolism, CGA: Comprehensive geriatric assessment, CABG: Coronary artery bypass grafting, DEX: Dexamethasone, ACEIs : Angiotensin-converting enzyme inhibitor, ARBs: Angiotensin II type 1receptor blockers. * SR did not report if elective &/or emergency surgery.

**Table S6:** **Traffic-light plots to summarise the overall effectiveness of Exercise/Physical activity interventions in any type of surgery**

**Table S6**: Traffic light plots of effectiveness of Exercise/physical activity interventions. 6MWT: 6 min walk test, w/e PT: Weekend Physical therapy, del: delayed, ADL: Activities of daily living, LoS: Length of stay, Ad to n/home: admission to nursing home, Postop comp: Postoperative complications, CABG: Coronary artery bypass grafting. * SR did not report if elective &/or emergency surgery.

**Table S7**: **Traffic-light plots to summarise the overall effectiveness of Diet/Nutritional interventions in any type of surgery**

**Table S7**: Traffic light plots of effectiveness of Diet/Nutritional Interventions. Supp: Supplements, Infect Comp: Infectious complications, Total comp: Total complications, ICU: Intensive care unit, Blood trans: Blood transfusion, ONS: Oral nutritional supplements, Admin: administration, AAs: Amino acids, GI: Gastrointestinal, LoS: Length of stay, SSI: Surgical site infection, +/- : with or without, PUFAs : polyunsaturated fatty acids. * SR did not report if elective &/or emergency surgery

**Table S8**: **Traffic-light plots to summarise the overall effectiveness of Care bundles, Multi-modal and Other interventions in any type of surgery**

**Table S8**: Traffic-light plots of effectiveness of care bundles, multi-modal and other interventions. POSH: Perioperative optimisation of senior health, CGA: Comprehensive geriatric assessment, LIFE: Liaison Intervention in frail elderly, LoS; length of stay, SSI: Surgical site infection, RBC: Red blood cell, Phys Funct: Physical Function, Coh St: Cohort studies, Total Comp: Total complications, Pul comp; Pulmonary complications, PPCs: Post operative pulmonary complications, NSAIDs: Non-steroidal anti-inflammatory drugs, TENS: Transcutaneous electrical nerve stimulation, p/way: pathway, POD: Postoperative delirium, Incid del: Incidence of delerium, sens anal: sensitivity analysis, S & L/term: Short and long term, ITS: Interrupted time series studies, S/ficial: Superficial, PTCA Percutaneous Transluminal Coronary Angioplasty, LC; laparascopic cholecystectomy. * SR did not report if elective &/or emergency surgery

**Table S9:** **Traffic-light plots to summarise the overall effectiveness of ERAS interventions in any type of surgery**

**Table S9**: Traffic-light plots of effectiveness of ERAS/FTS interventions. ERAS: Enhanced recovery after surgery, FTS: Fast track surgery, AA: aortic aneurysm, THA : Total hip arthroplasty, TKA: Total knee arthroplasty, O/paed: Orthopaedic surgery, Gastric C surg: Gastric cancer surgery, Overall comp: overall complications, GI: Gastrointestinal, VAS: Visual analogue scale, OFB amb: Time to out of bed ambulation , POI: postoperative paralytic ileus, EN: Enhancement of nutrition , PPOI: prolonged postoperative ileus. Min Invas: Minimally invasive. * SR did not report if elective &/or emergency surgery
